# Under-detection of endospore-forming *Firmicutes* in metagenomic data

**DOI:** 10.1016/j.csbj.2015.04.002

**Published:** 2015-04-25

**Authors:** Sevasti Filippidou, Thomas Junier, Tina Wunderlin, Chien-Chi Lo, Po-E Li, Patrick S. Chain, Pilar Junier

**Affiliations:** a Laboratory of Microbiology, Institute of Biology, University of Neuchatel, CH-2000, Neuchâtel, Switzerland; b Vital-IT group, Swiss Institute of Bioinformatics, CH-1015 Lausanne, Switzerland; c Bioscience Division, Los Alamos National Laboratory, Los Alamos, NM 87545, USA

**Keywords:** Endospores, *gpr*, Metagenomics, Profile analysis, *spo0A*

## Abstract

Microbial diversity studies based on metagenomic sequencing have greatly enhanced our knowledge of the microbial world. However, one caveat is the fact that not all microorganisms are equally well detected, questioning the universality of this approach. *Firmicutes* are known to be a dominant bacterial group. Several *Firmicutes* species are endospore formers and this property makes them hardy in potentially harsh conditions, and thus likely to be present in a wide variety of environments, even as residents and not functional players. While metagenomic libraries can be expected to contain endospore formers, endospores are known to be resilient to many traditional methods of DNA isolation and thus potentially undetectable. In this study we evaluated the representation of endospore-forming *Firmicutes* in 73 published metagenomic datasets using two molecular markers unique to this bacterial group (*spo0A* and *gpr*). Both markers were notably absent in well-known habitats of *Firmicutes* such as soil, with *spo0A* found only in three mammalian gut microbiomes. A tailored DNA extraction method resulted in the detection of a large diversity of endospore-formers in amplicon sequencing of the 16S rRNA and *spo0A* genes. However, shotgun classification was still poor with only a minor fraction of the community assigned to *Firmicutes*. Thus, removing a specific bias in a molecular workflow improves detection in amplicon sequencing, but it was insufficient to overcome the limitations for detecting endospore-forming *Firmicutes* in whole-genome metagenomics. In conclusion, this study highlights the importance of understanding the specific methodological biases that can contribute to improve the universality of metagenomic approaches.

## Highlights

• Endospore formers were under-detected by profile analysis of sporulation genes in metagenomes.
• Endospore formers were absent even from those habitats known to harbor them.
• A tailored DNA extraction method improved detection in amplicon sequencing.
• Ameliorated DNA extraction did not improve shotgun classification.
• Endospore-formers represent an undetectable community fraction by metagenomic approaches.

## 1 Introduction

Metagenomic studies have emerged as promising methods for the collective study of microbial communities directly extracted from environmental samples [1–3]. These approaches have been successfully applied to a variety of environments and have helped to unveil new functional pathways and metabolic processes within the microbial world [4–8].

Biases, however, can occur at all the steps involved in a metagenomic workflow. They can be associated to the specific type of environment [9,10], the DNA yields obtained [11], the DNA extraction method [12], the amplification (for example in amplicon sequencing), but also in the sequencing and the analysis of the sequences. These limitations have been highlighted in the recent literature and result in problems such as low coverage of the less abundant taxa (the so-called “depth bias” for example in the detection of ribosomal genes [13]), low reproducibility of results [14] and underrepresentation of certain taxa, as discussed herein. In order to overcome these limitations, new approaches have been developed including single-cell genomics or culture-dependent methodologies such as culturomics [15,16] which, in their turn, have their own limitations.

Even though methodological bias of metagenomic diversity surveys associated to particular types of environments such as soil has been demonstrated experimentally [9,10], the specific coverage of individual microbial groups within the community is still unknown. One example of a bacterial group that can be used to test coverage bias in metagenomic datasets is endospore-forming *Firmicutes*. Even though, culturing of microorganisms is largely acknowledge to be biased, according to previous research based on culture collections as well as whole-genome sequencing, *Firmicutes* is the second most abundant bacterial phylum [17]. Endospore formers live in a wide range of environments on Earth's surface and subsurface [18,19]. The hardy outer cortex of endospores and the small acid–soluble proteins stabilizing their DNA [20–22], allow these bacteria to be distributed into every habitat on Earth [23]. However, a phylogenetic assessment of the microbial communities in four metagenomic datasets has revealed surprisingly few endospore formers [24]. This might appear surprising considering their ubiquity, but endospores are known to withstand many traditional methods of DNA isolation and are thus potentially undetectable in a sample. Recently, a DNA extraction method for the extraction of resistant structures such as endospores has been developed by our group [12]. This DNA extraction method was combined with amplicon sequencing of the gene coding the master regulator for the initiation of sporulation (*spo0A* gene) to demonstrate an improved detection of endospore-forming *Firmicutes* in sediment samples [12]. Our group has developed further methods to separate endospores from vegetative cells, which has open the possibility to carry out genomic studies only focused on endospores [12,25]. These two studies demonstrate by amplicon sequencing that the diversity of endospore-forming *Firmicutes* is far from uncovered. However, the effectiveness of the improved DNA extraction method for whole-genome metagenomic studies is unknown.

The aim of this study was to measure the level of detection of endospore formers in metagenomic studies carried out so far, and to evaluate the effect of an improved DNA extraction method on the detectability of this group. To do this, we initially searched for functional gene markers of endospore formation in metagenomic datasets using profiles. We then applied a modified DNA extraction method that is tailored to release DNA from resistant structures such as endospores [12] in a selected environmental sample. Amplicon sequencing of the 16S rRNA and *spo0A* genes were performed on the sample in order to assess the relative abundance and phylogenetic diversity of *Firmicutes*. This was complemented by shotgun sequencing and classification of the metagenome reads. Our results indicate that endospore-forming *Firmicutes* are overlooked in environmental diversity surveys using traditional whole metagenomic approaches.

## 2 Materials and Methods

### 2.1 Genome Sequence Retrieval

Complete and draft genome sequences of endospore-forming *Firmicutes* were downloaded from the Comprehensive Microbial Resource (CMR, 24.0 data release, cmr.jcvi.org) and Integrated Microbial Genomes (IMG, 3.0, img.jgi.doe.gov) websites. Protein and nucleotide sequences of spore-related genes were obtained by search for role category/function *sporulation and germination* (CMR) and sporulating (IMG). Additional information on all retrieved genomes was obtained from the GenBank database (www.ncbi.nlm.nih.gov/genome).

### 2.2 Detection of Orthologous Sporulation Genes Common to All Endospore-Formers

Orthologous groups were delineated based on best reciprocal BLASTp hits [26]. BLASTp was used to align each sequence in the set against all sequences except those of the same species (thus avoiding paralogs). The best hit in each species was retained, and sequence pairs, that were each other's best match, were defined as best reciprocal hits (BRHs). Putative orthologous groups were defined using the algorithm used by OrthoDB [27]. OrthoDB has data on Fungi, Metazoa, and Bacteria. An early version of the BRHCLUS program (unpublished at the time) was obtained from its author, Dr. Tegenfeldt (pers. comm) and run according to the author's instructions. The program is now available from http://orthodb.org/. To our knowledge, its utility does not depend on the clade it is used for — OrthoDB uses the same clustering program for all data in its scope.

### 2.3 Profile Construction and Validation

The genomic sequences were filtered in such as way as to keep only one (randomly chosen) sequence per genus, thus reducing taxonomic sampling bias. Multiple alignments of Spo0A and Grp were produced with MAFFT [28]. Gribskov-style sequence profiles were constructed with EMBOSS's prophecy program [29]. The profiles’ score cutoffs were determined by searching with EMBOSS's prophet program against the original Spo0A (resp. Gpr) sequence set as a positive control, and against shuffled versions of the same as negative set.

### 2.4 Metagenomic Datasets Retrieval

The metagenome datasets (supplementary Table 1) were downloaded from IMG, GOLD (genomesonline.org), or the metagenomes subset of the WGS section of EMBL (ebi.ac.uk/genomes/wgs.html). These datasets included all the metagenomic studies available at EMBL when the profile analysis was performed. Only sequences or contigs of > 800 bp, which are slightly shorter than the full-length sporulation genes, were kept for analysis.

### 2.5 Environmental Sampling, DNA Extraction and Quantitative PCR

The sample was collected at Nea Apollonia (NAP) geothermal spring (N 40° 39,191′ E 22° 56,707′), Greece, in June 2011. Geothermal reservoir was reached through a 120 m drilling pipe, used mostly for pumping 80 °C water for bathing purposes. Biofilm from the pipe interior was collected and frozen within 2 h of collection. Upon arrival at the laboratory, a tailored DNA extraction method previously described [12] was applied to the sample. More precisely, DNA was extracted using the FastDNA Spin Kit for Soil (MP Biomedicals, California), using a modified protocol in order to ensure that DNA was not only extracted from vegetative cells but also from spores and other cells difficult to lyse. These modifications were (a) a separation of the biomass from the soil, using a Na-hexa-meta-phosphate solution and (b) a sequential bead-beating step (three times) to ensure mechanical disruption of cells. In total, 10ug of high molecular RNA-free DNA was obtained.

Moreover, 16S rRNA gene and *spo0A* gene copy numbers were calculated using a quantitative PCR assay, as previously described [30].

### 2.6 Amplicon Sequencing of the 16S rRNA and *spo0A* Genes

In order to verify the presence and relative abundance of endospore formers, 454 pyrosequencing of a fragment of the 16S rRNA and *spo0A* genes was firstly applied to the sample NAP. Sequencing was done using the services of Eurofins MWG Operon (Ebersberg, Germany). For 16S rRNA amplicon sequencing, fragments of approximately 500 bp were retrieved using primers Eub8f (5′-AGAGTTTGATCCTGGCTCAG-3′) and Eub519r (5′-GTATTACCGCGGCTGCTGG-3′), as previously described [31]. 16S rRNA gene raw sequence data was analyzed with QIIME [32], using the pipeline for de novo OTU picking. OTUs were identified using a threshold of 97% sequence similarity. The sequences were then clustered into putative OTUs with the pick_otus.py program from the QIIME package using the Uclust method [32]. The single sequence picked by the program as a representative of each OTU was used to build a phylogeny.

For the *spo0A* amplicon sequening, a 602 bp sequence of the *spo0A* gene was amplified using the degenerated primer *spo0A*166f (5′-GATATHATYATGCCDCATYT-3′) and *spo0A*748r (5′-GCNACCATHGCRATRAAYTC-3′) [12]. 42′151 sequences were received from the sample. Sequences were then filtered according to Phred [33] quality score (minimum of 30) and sequences of length shorter than 600 bp were removed. Remaining sequences were translated to their amino acid sequence; resulting full-length ORFs were then matched against the *spo0A* profile, in order to confirm that the primers actually amplified the *spo0A* sequences.

Phylogenies were constructed from Phylip-formatted alignments with PhyML [34], using default parameters. The trees were re-rooted, condensed according to protocol, and displayed with the Newick Utilities [64]. Each branch represents a cluster of OTUs of > 97% sequence similarity. Identification of the closest relatives of the environmental sequences was done by protein BLAST [26] with the translated protein sequences using a reference database of 581 *spo0A* protein sequences from the InterPro site [35].

All metagenomic sequences were submitted to GenBank. The 16S rRNA amplicon sequencing data can be retrieved under the BioProject ID PRJNA267761 and BioSample ID SAMN03198953 and the *spo0A* amplicon sequencing data under the BioProject ID PRJNA276803 and Biosample ID SAMN03392534.

### 2.7 Metagenomic Sequencing

Once high prevalence of endospore formers was confirmed in the 16S rRNA pyrosequencing data (41% of total bacterial community), whole-metagenome sequencing of NAP was performed on a full plate of a GS FLX platform, followed by de novo assembly using the services of GATC- biotech (Konstanz, Germany). The metagenome dataset can be retrieved from GenBank under the BioProject ID PRJNA271123 and BioSample ID SAMN03273062.

### 2.8 Metagenome Data Annotation

Several tools were used to produce the read-based metagenomic analysis of NAP metagenome dataset. GOTTCHA [36] was run using BWA [37] against 4 databases consisting of Phylum, Genus, Species and Strain-level unique signatures. MetaPhlAn v1.7.7 [38] was run using BowTie2 [39] with default parameters against its clade-specific maker genes database. Kraken was run with its reduced taxonomic-specific 31-mer database (mini-database). BWA v0.7.4-r385 used as a stand-alone tool was run locally using BWA-backtrack algorithm to map reads against a custom database of bacterial, archaeal and viral complete genomes retrieved from NCBI RefSeq database [40]. The mapped reads were subsequently assigned to organisms by mapping the GI numbers of aligned references to NCBI taxonomic ID and rolled up to higher ranks. mOTUs v1.0 [41] was run with the database composed of 10 universal marker genes and LMAT v1.2.1 [42] was run with the pre-computed reference search database (kML.18mer.16bit.reduced.db) with default parameters. Since BWA (standalone), Kraken and LMAT only reported read counts of taxonomies, the relative abundances were represented by the portion of total classified reads in these tools. While each tool tries to identify similarities among the reads and the databases used, each tool is centered around a different algorithmic approach to solve this complex challenge, using either a unique search algorithm, a uniquely designed database, or both. The interpretation of the results from each tool should thus be taken within its own context. For example, mOTUs and MetaPhlAn use pre-selected marker genes to perform the analysis, however different marker genes are used and different methods are used to identify reads that are similar to these marker genes. Kraken and LMAT both use subsequences within reads (k-mers) and match k-mers observed within the reads with those observed within known reference genomes. Meanwhile BWA is a read-mapping tool that we use against the refseq database to report matching reads.

## 3 Results and Discussion

### 3.1 Selection of Functional Markers for Endospore-formation

We recently identified functional marker genes involved in endospore formation in endospore-forming *Firmicutes* [12]. Bidirectional BLAST of the genes annotated as part of the cellular function of sporulation allowed to select six highly conserved orthologous genes as part of the endospore-forming *Firmicutes* proteome. Among those, *spo0A* and *gpr*, were selected for the construction of profiles based on their consistent phylogenetic reconstruction with the 16S rRNA gene phylogeny. These two genes represent significant stages of the endospore-formation process, namely the commitment to enter sporulation (*spo0A*) and the proteolytic activity on acid-soluble spore proteins (SASPs) during germination (*gpr*) [43]. In recent studies analyzing the minimal set of endospore-formation genes required by endospore-formers had indicated that *spo0A* is indeed one of the most conserved genes almost exclusively found among this bacterial group [44–46]. In the case of *gpr*, it has been shown that it belongs to a category of genes present in *Bacillus* and *Clostridium* without any known ortholog in Gram-negative Proteobacteria or Cyanobacteria [21].

### 3.2 Profile Analysis of Sporulation Genes in Metagenomes

**Fig. 1:**
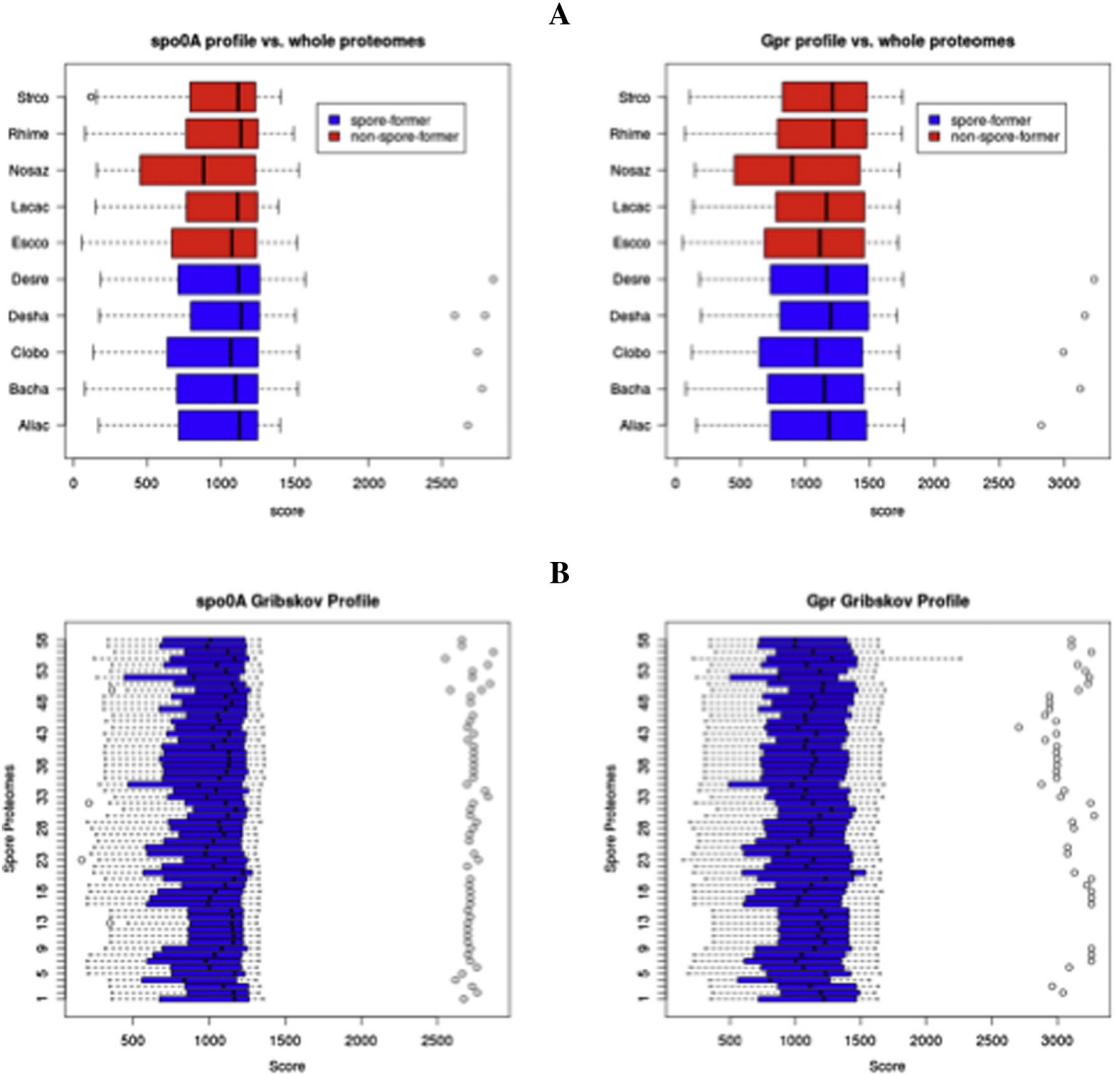
A. Validation of the profiles created for the genes *spo0A* and *gpr* compared to a selection of genomes of endospore-forming *Firmicutes* (blue bars) and non spore-forming genomes (red bars). In endospore-forming *Firmicutes* a single hit with a score above 2000 (Spo0A) and 2500 (Gpr) distinguish between positive and negative hits. Strco = *Streptomyces coelicolor*; Rhime = *Rhizobium melliloti*; Nosaz: *Nostoc azollae*; Lacac = *Lactobacillus acidophilus*; Escco = *Escherichia coli*; Desre = *Desulfotomaculum reducens*; Desha = *Desulfitobacterium hafniense*; Clobo = *Clostridium botulinum*; Bacha = *Bacillus halodurans*; Aliac = *Alicyclobacillus acidocaldarius*. B. The same analysis was repeated using all 59 endospore-forming genomes retrieved from IMG and CMR databases (see supplementary Table 1).

Profiles of Spo0A and Gpr were constructed and compared to metagenomic datasets to find sequences of high similarity with *spo0A* and *gpr*. Profiles are models of conserved sequences built from an alignment and are more sensitive than BLAST or other pair-wise comparisons especially for protein searches [47]. The sequence profiles were generated based on 14 aligned sequences. They were validated on genomes of known endospore-forming and non-sporulating bacteria (Fig. 1A). A single positive hit was found in the genome of each endospore-forming bacterium, while no hits were found in the negative controls. This result also allowed determining a score cut-off for *sp*o0ASpo0A (2000) and Gpr (2500) profiles to distinguish between positive and negative hits. Using this cut-off value one orthologous sequence of each of the two genes could be detected in a further 59 genomes of endospore-forming bacteria (Fig. 1B) reported in the genomic databases of the Comprehensive Microbial Resource (CMR) and Integrated Microbial Genomes (IMG) (Supplementary Table 1).

The profile analysis was then used to detect Spo0A or Gpr in publicly available environmental metagenomes. For this, 73 microbial metagenomic datasets (Supplementary Table 2) from a total of 25 publications or direct submissions were retrieved. The datasets consisted of 6,220,494 sequences of average length of 957 bp and represented different environments, including marine, fresh- and ground-waters, acid mine drainage, compost, hypersaline environments, hot springs, soils, sludge, food and organism-associated environments (ant fungus garden, coral, fish and human gut).

**Fig. 2:**
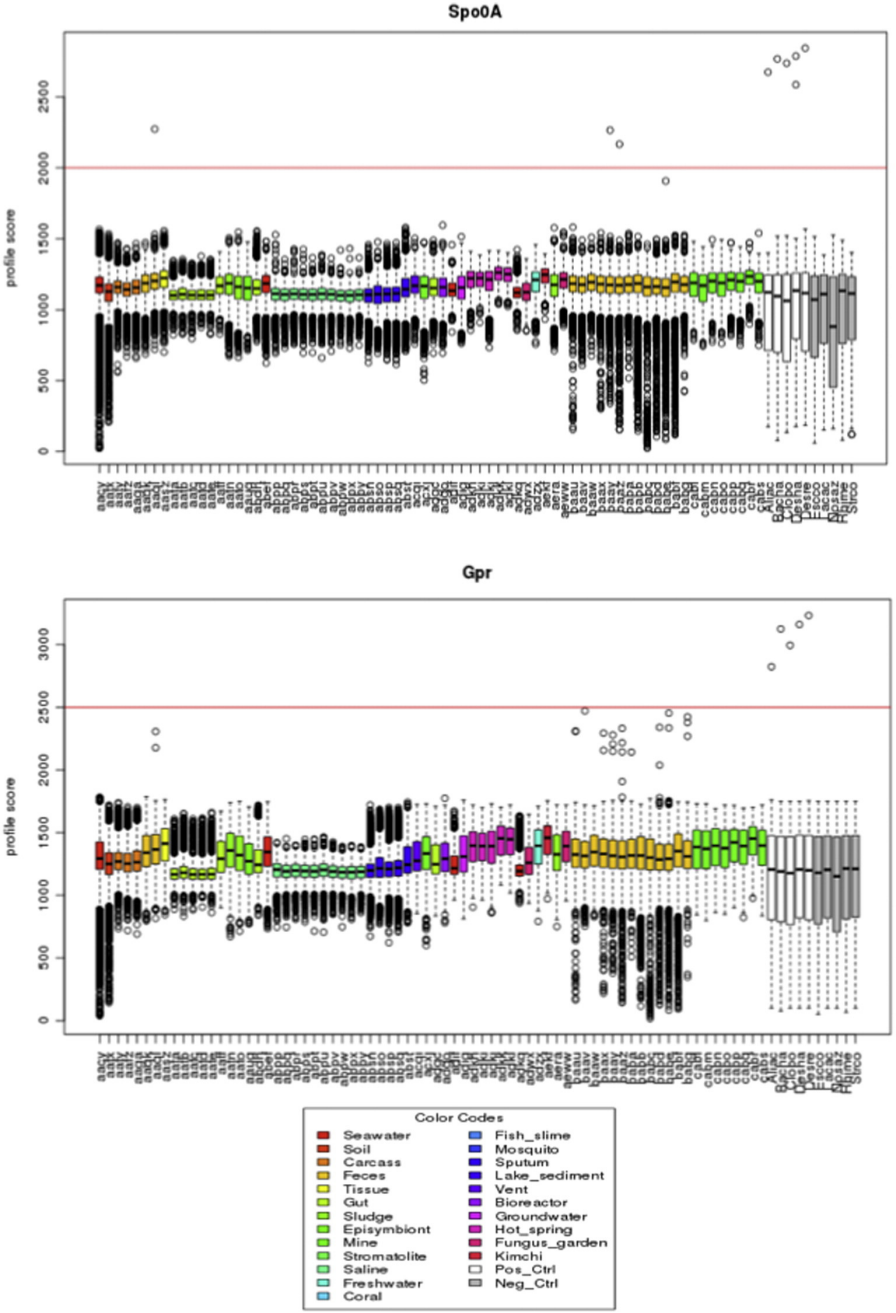
Profile similarity hits for Spo0A and Gpr protein profiles in metagenomes from different origins. The color code identifying different environments is presented under the results. The genomes included in profile testing (see Fig. 1A) were also included in the analysis and are presented in white (endospore-formers) and gray (non-spore formers).

The profile analysis revealed only three sequences with a score above the cutoff of the Spo0A profile in all metagenomic datasets (Fig. 2A). All three metagenomes (AAQL, BAAY, BAAZ) originated from human gut [48,49], in which *Firmicutes* are known to be one of the dominant bacterial groups [50,51]. For the *gpr* gene profile (Fig. 2B), no sequences were found with a similarity score above the cutoff value. These results are surprising considering that some of these metagenomes were sampled in environments with high abundance of endospore-forming *Firmicutes* (e.g. gut or soil; [52,53]). These results showed that these two genes from endospore-forming *Firmicutes* are underrepresented in metagenomes. This had been alluded to earlier by von Mering et al., [24], and is now confirmed here.

A methodological bias during the DNA extraction of resistant structures such as bacterial endospores has been suggested as the origin of an underrepresentation of microbial groups producing this structure [24]. Indeed, independently of the methodological approach taken (i.e. whole genome shotgun analysis, activity- or sequence-driven screening), the first and most crucial step in any metagenomic project is the extraction of nucleic acids. The isolated DNA should be representative of all cells in the sample and of sufficient quality and amount for subsequent sequencing [54]. Clearly, not all microbial species are equally amenable to the DNA extraction methods used today [9,10], especially considering the diversity of morphological and physiological states in which microbes can be found in environmental samples. Therefore, complementary information, in particular concerning the method used for DNA extraction of the metagenomes was thus considered. The described DNA extraction methods (Supplementary Table 2) consisted of enzymatic or chemical protocols (18 datasets) or mechanical procedures of cell lysis (8 datasets). Sequences associated to *Firmicutes* are reported for some of the analyzed metagenome projects regardless of the DNA extraction protocol. For example, sequences of Clostridia (30%) and Bacilli (1%) were reported in the wallaby gut extracted enzymatically [55]. Also, in the compost metagenome extracted by bead beating, more than 13% of sequences were reported as members of endospore-formers *Bacillus* spp. or *Paenibacillus* spp. [56]. Our profile analyses however, do not show positive hits for Spo0A and Gpr in either of these metagenomes. Whether this is due to the extraction method applied, to the depth of sequencing or to other specific bias is hard to establish.

We have developed a tailored DNA extraction method that allows a better assessment of the abundance and diversity of endospore-formers in environmental samples for amplicon sequencing [12,57]. Therefore, we next evaluated if using this extraction protocol in an environmental sample could improve the detection of endospore-formers in a metagenome.

### 3.3 Amplicon Sequencing of an Environmental Sample With High Prevalence of Endospore-forming *Firmicutes*

**Fig. 3:**
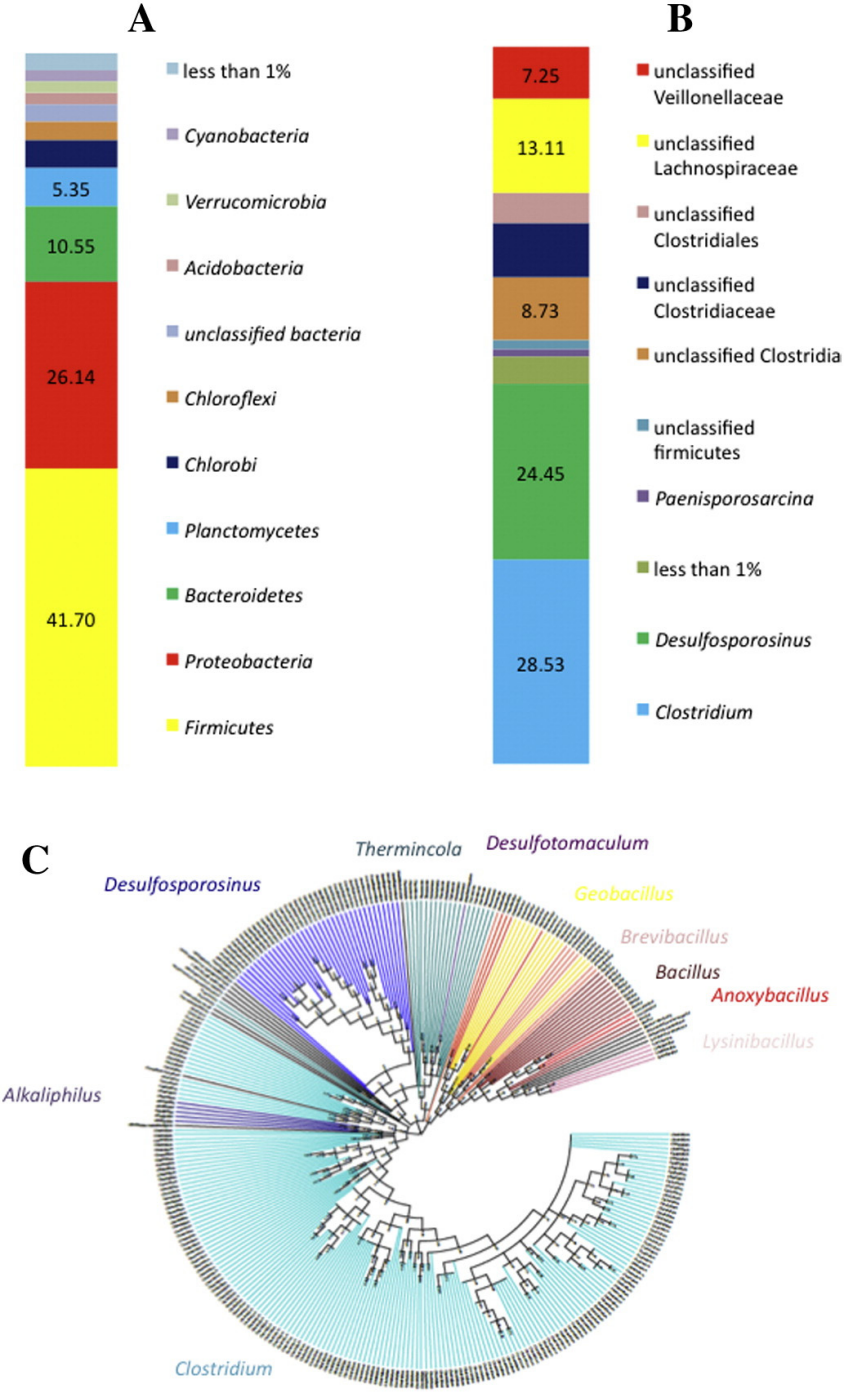
Analysis of pyrosequencing results obtained from 16S rRNA gene and *spo0A* amplicons, from an environmental sample with high prevalence of endospore-forming *Firmicutes* (Nea Apollonia, NAP). (A) Total 16S rRNA gene community composition to the phylum level. (B) Firmicute fraction of the total community (16S rRNA gene) to the genus level. (C). Cladogram representing the community composition of *Firmicutes* using the *spo0A* gene. Sequences color coded by genus.

We performed amplicon sequencing from a sample in which high prevalence of endospore-forming *Firmicutes* was suspected from the ratio of 16S rRNA (bacterial) and *spo0A* (endospore-formers) gene numbers measured by quantitative PCR [58]. This ratio was obtained from DNA extracted using our modified protocol. Sequencing of the 16S rRNA and *spo0A* gene amplicons was conducted and revealed not only a high prevalence of endospore-forming *Firmicutes*, but also a high diversity of endospore formers (Fig. 3).

In the amplicon sequencing of the 16S rRNA gene, *Firmicutes* accounted for 41.70% of the total bacterial community. The abundance of 16S rRNA amplicons corresponding to *Firmicutes* was nearly double the amount of Proteobacteria, which was the second most abundant bacterial Phylum (26.14%). Among the endospore-formers observed in the pyrosequencing results, the genera *Clostridium* and *Desulfosporosinus* dominated the community in the sample, indicating a clear dominance of anaerobic endospore-formers [59] as could be expected considering the temperature and other environmental conditions at this geothermal spring. Amplicons affiliated to *Clostridium* and *Desulfosporosinus* were also dominant in the *spo0A* amplicon sequencing, which also showed the dominance of anaerobic endospore-formers. Even though *spo0A* sequences related to aerobic endospore-formers (e.g. *Geobacillus* and *Bacillus*) were also obtained, the classification of the *spo0A* from aerobic endospore-formers was ambiguous as shown by the existence of, for example, clades related to *Anoxybacillus* but placed at different positions in the phylogeny (Fig. 3C). In fact, only recently environmental *spo0A* sequences have started to be obtained [12], and the phylogenetic assignment needs to be refined.

### 3.4 Metagenomic Sequencing

In addition to pyrosequencing, the same sample was also subjected to metagenomic sequencing. It is worth mentioning that in whole-genome metagenomics a PCR amplification bias does not apply and thus we did not necessarily expect to find the same groups or the same frequency detected in the amplicon sequencing. However, the results of the qPCR quantification and the amplicon sequencing were taken as an indication of the prevalence of *Firmicutes* in this specific environmental sample. The NAP dataset consisted of a total of 481,810 sequences of average length of 330 bp. When the Spo0A and Gpr profile analyses were conducted on this metagenome, none of the two genes were detected. However, looking only at two specific genes could be an issue, since those could be, for various reasons, underrepresented in the sequences. Therefore, an extended search for reads that could be assigned to *Firmicutes* using different prediction tools on the assembled metagenome was also carried out.

Relative abundances from classified reads were considered to establish the five most prevalent Phyla present in the sample (Table 1). *Firmicutes* appear in the top five Phyla only for two of the four prediction tools used. In the case of Kraken, *Firmicutes* reads corresponded to 1.60% of the classified data, being the third most abundant phylum (the most abundant one was Proteobacteria with 82.71%). BWA predicted 5.32% of the classified sequences as to belong to *Firmicutes* (second most abundant phylum after Proteobacteria with 75.21%). *Firmicutes* were not listed after classification with MetaPhlAN and LMAT. Likewise, when reconstruction of full bacterial genomes was attempted for the NAP metagenome using MetaPhlAn, none of the top 5 microorganisms was assigned to *Firmicutes* (data not shown).

Thus, even though amplicon sequencing revealed a large fraction of the community as belonging to *Firmicutes*, this was not observed in the shotgun metagenome. There are several possible explanations for these results. One of those is the fact that the ribosomal (rrn) operon is normally found in several copies and thus the representation of a microbial community based on 16S rRNA gene sequencing is skewed. Furthermore, the average number of rrn operon copies depends on the group of bacteria. An average value of 7.01 copies of 16S rRNA genes was found for the phylum *Firmicutes* in the rrnDB [60], which implies that this group can be overrepresented in 16S rRNA gene amplicon libraries. In addition, it should be noted that for all the tools used, classification was poor and only a very small fraction of the sequences could be actually assigned to a particular taxonomic group. Therefore, the lack of detection of *Firmicutes* could be due to the current limitations of the analysis tools. In fact, recent sequencing technologies generate such large quantities of data as to bring along a new set of challenges in data analysis, the so-called bioinformatics bottleneck [61]. On the level of interpretation of metagenomic data there is still an important amount of unexplored information available from the results, simply because the advances in sequencing technologies are greater than the complementary progress in annotation, data inventory and standardization of metadata [14].

## 4 Conclusions

Since Staley and Konopka introduced the “great plate count anomaly” [62,63], revealing that only a small fraction of the microbial community can be cultured in the laboratory, one of the great challenges in environmental microbiology is the understanding of the diversity and metabolic capabilities of microbes in a culture-independent manner. That bias was partly overcome by moving into the direction of directly extracting genetic material from environmental samples. However, our results reveal that for specific microbial groups, we are still in a phase in which, similar to a percentage of the community being *not culturable* in culture-based approaches, a fraction of the genomes of the community might be considered as *not detectable* for culture-independent approaches. Nonetheless, profiling of the taxonomic and phylogenetic composition of microbial communities is at the heart of many metagenomic studies, and it is an obligatory step to draw conclusions on the role of microorganisms in the environment based on metagenomics. Our results suggest that in the case of endospore-forming *Firmicutes*, classification by various methods still lags behind. However, starting from samples such as NAP, in which evidence for high frequency of this bacterial group exists, could be the first step towards developing improved methods of classification and phylogenetic assignment of metagenomic data.

## Figures and Tables

**Table 1:** Prevalence of *Firmicutes* in 16S rRNA gene amplicon sequencing and shotgun metagenomic sequencing applied to the NAP sample. Different prediction tools were used to establish the five most frequent Phyla in the samples. With the exception of the 16S rRNA gene amplicon sequencing, the relative percentage indicated corresponded to the fraction of the sequences that could be classified and not to the frequency of any of the groups for the total reads generated after sequencing.

Prediction tool	Top 5 Phyla		Frequency	Relative %
16S RNA gene amplicon pyrosequencing (QIIME)	1	*Firmicutes*	41.70	41.70%
	2	Proteobacteria	26.14	26.14%
	3	Bacteroidetes	10.55	10.55%
	4	Planctomycetes	5.35	5.35%
	5	Chlorobi	3.88	3.88%
Kraken (mini database)	1	Proteobacteria	16644	82.71%
	2	Actinobacteria	1744	8.67%
	3	*Firmicutes*	322	1.60%
	4	Bacteroidetes	298	1.48%
	5	Cyanobacteria	192	0.95%
MetaPhlAn	1	Proteobacteria	82.01061	82.01%
	2	Chloroflexi	9.24158	9.24%
	3	Actinobacteria	2.32449	2.32%
	4	Bacteroidetes	2.08071	2.08%
	5	Acidobacteria	1.54098	1.54%
BWA	1	Proteobacteria	452	75.21%
	2	*Firmicutes*	32	5.32%
	3	Thaumarchaeota	28	4.66%
	4	Actinobacteria	26	4.33%
	5	Bacteroidetes	17	2.83%
LMAT	1	Ascomycota	425	35.68%
	2	Cyanobacteria	385	32.33%
	3	Proteobacteria	190	15.95%
	4	Thaumarchaeota	145	12.17%
	5	Basidiomycota	20	1.68%
