# Supplementary material for: Under-detection of endospore-forming Firmicutes in metagenomic data
Source: Comput Struct Biotechnol J. 2015 Apr 25;13:299–306. doi: 10.1016/j.csbj.2015.04.002 (PMC4427659; doi:10.1016/j.csbj.2015.04.002)
Supplement: Supplementary file 1 — Supplementary Tables 1 and 2. [file mmc1.docx]

**A cautionary tale of under-detected endospore-forming Firmicutes in metagenomic data**

Sevasti Filippidou^1^, Thomas Junier^1,2^, Tina Wunderlin^1§^, Chien-Chi Lo^3^, Po-E Li^3^, Patrick S. Chain^3^, Pilar Junier^1*^

^1^Laboratory of Microbiology, Institute of Biology, University of Neuchatel, CH-2000, Neuchâtel, Switzerland

^2^Vital-IT group, Swiss Institute of Bioinformatics, CH-1015, Lausanne, Switzerland

^3^Bioscience Division, Los Alamos National Laboratory, Los Alamos, New Mexico, 87545, USA

**Supplementary Information**

**Supplementary Table 1.** Complete genome sequences from 57 endospore-forming bacteria used in this study. All genomes were retrieved from the CMR database.


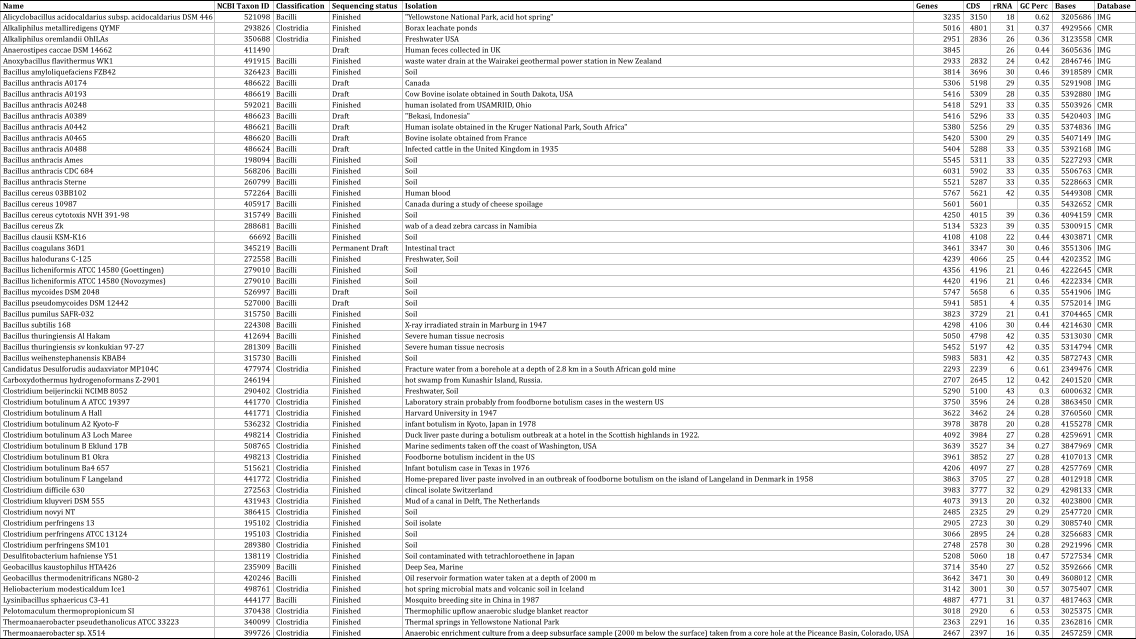


**Supplementary Table 2.** Metagenomic datasets used to test the presence of endospore-forming bacteria in environmental samples by profile analyses with Spo0A and Gpr. Grouping types of DNA information was used for the color code in Figure 1. For all direct submissions (Reference column) the cell lysis method was not indicated in the submission. NA= the information on the percentage of Firmicutes in the dataset is not available.

| **Accession Code** | **Grouping types of DNA** | **Cell lysis method** | **Positive hit Spo0A** | **Firmicutes abundance (%)** | **Reference** |
| --- | --- | --- | --- | --- | --- |
| ADGO | Compost | Bead-beating |  | *Bacillus* sp. 7%, *Bacillus* sp. 5%, *Paenibacillus* sp. 1%, | (Allgaier et al 2010) |
| AEWW | Ant fungus garden | NA |  | NA | Aylward et al, Direct submission |
| CABL, CABM, CABN, CABO, CABP, CABQ, CABR, CABS | Mine drainage | Vortex |  | NA | (Bertin et al 2011) |
| ACQI | Hydrothermal vent | Mortar |  | NA | (Brazelton and Baross 2009) |
| ACXJ | Mine drainage | Enzyme, chemical |  | NA | (Dick et al 2009) |
| ADZX | Freshwater | Enzyme, chemical |  | Firmicutes 5% | (Ferrer et al 2011) |
| AAQK, AAQL | Gut | Bead-beating | yes | 16S: Firmicutes > 85% | (Gill et al 2006) |
| AAUQ | Epibiont | Enzyme, chemical |  | NA | (Grzymski et al 2008) |
| ADIG | Groundwater | Mortar, freeze-thaw |  | *Bacilli* 0.12% | (Hemme et al 2010) |
| ADKH, ADKI, ADKJ, ADKK, ADKL | Hot Springs | NA |  | NA | Inskeep et al, 2010, Direct submission |
| AEKF | Food | Bead-beating |  | NA | (Jung et al 2011) |
| ABSN, ABSO, ABSP, ABSQ, ABSR | Freshwater | Enzyme, chemical |  | NA | (Kalyuzhnaya et al 2008) |
| ABEF | Marine | Enzyme, chemical |  | NA | (Konstantinidis et al 2009) |
| ABPP, ABPQ, ABPR, ABPS, ABPT, ABPU, ABPV, ABPW, ABPX, ABPY | Hypersaline mat | Vortex |  | NA | (Kunin et al 2008) |
| BAAU, BAAV, BAAW, BAAX, BAAY, BAAZ, BABA, BABB, BABC, BABD, BABE, BABF, BABG | Gut | Enzyme, chemical | yes | NA | (Kurokawa et al 2007) |
| ADIF, ADKQ | Marine | NA |  | NA | Lucas et al, 2010, Direct submission |
| ADGC | Gut | Enzyme, chemical |  | *Clostridia* 30%, *Bacilli* <1% | (Pope et al 2010) |
| AERA | Sludge | NA |  | NA | Purohit et al, 2011, Direct submission |
| ADWX | Ant fungus garden | Physical |  | NA | (Suen et al 2010) |
| AAFX, AAFY, AAFZ, AAGA | Soil, Whale fall | Enzyme, chemical |  | NA | (Tringe et al 2005) |
| AATA, AATB, AATC, AATD, AATE, AATF | Gut | Physical |  | NA | (Turnbaugh et al 2006) |
| AACY | Marine | Enzyme, chemical |  | 0.5% Firmicutes (clones) | (Venter et al 2004) |
| ABDH | Gut | Enzyme, chemical |  | 7% Firmicutes (16S based) of total of 216 OTUs | (Warnecke et al 2007) |
| AASZ | Oligochaete symbionts | Enzyme, chemical |  | NA | (Woyke et al 2006) |
| AATN, AATO | Sludge | Bead-beating |  | NA | (Martin et al 2006) |

References

Allgaier M, Reddy A, Park JI, Ivanova N, D'Haeseleer P, Lowry S *et al* (2010). Targeted Discovery of Glycoside Hydrolases from a Switchgrass-Adapted Compost Community. *Plos One* **5**.

Bertin PN, Heinrich-Salmeron A, Pelletier E, Goulhen-Chollet F, Arsene-Ploetze F, Gallien S *et al* (2011). Metabolic diversity among main microorganisms inside an arsenic-rich ecosystem revealed by meta- and proteo-genomics. *ISME J* **5:** 1735-1747.

Brazelton WJ, Baross JA (2009). Abundant transposases encoded by the metagenome of a hydrothermal chimney biofilm. *Isme Journal* **3:** 1420-1424.

Challacombe JF, Altherr MR, Xie G, Bhotika SS, Brown N, Bruce D *et al* (2007). The complete genome sequence of Bacillus thuringiensis Al Hakam. *J Bacteriol* **189:** 3680-3681.

Chen XH, Koumoutsi A, Scholz R, Eisenreich A, Schneider K, Heinemeyer I *et al* (2007). Comparative analysis of the complete genome sequence of the plant growth-promoting bacterium Bacillus amyloliquefaciens FZB42. *Nat Biotechnol* **25:** 1007-1014.

Chivian D, Brodie EL, Alm EJ, Culley DE, Dehal PS, Desantis TZ *et al* (2008). Environmental genomics reveals a single-species ecosystem deep within Earth. *Science* **322:** 275-278.

Dick GJ, Andersson AF, Baker BJ, Simmons SL, Yelton AP, Banfield JF (2009). Community-wide analysis of microbial genome sequence signatures. *Genome Biology* **10**.

Edgar RC (2004). MUSCLE: multiple sequence alignment with high accuracy and high throughput. *Nucleic Acids Res* **32:** 1792-1797.

Feng L, Wang W, Cheng J, Ren Y, Zhao G, Gao C *et al* (2007). Genome and proteome of long-chain alkane degrading Geobacillus thermodenitrificans NG80-2 isolated from a deep-subsurface oil reservoir. *Proc Natl Acad Sci U S A* **104:** 5602-5607.

Ferrer M, Guazzaroni ME, Richter M, Garcia-Salamanca A, Yarza P, Suarez-Suarez A *et al* (2011). Taxonomic and Functional Metagenomic Profiling of the Microbial Community in the Anoxic Sediment of a Sub-saline Shallow Lake (Laguna de Carrizo, Central Spain). *Microbial Ecology* **62:** 824-837.

Gill SR, Pop M, DeBoy RT, Eckburg PB, Turnbaugh PJ, Samuel BS *et al* (2006). Metagenomic Analysis of the Human Distal Gut Microbiome. *Science* **312:** 1355-1359.

Gioia J, Yerrapragada S, Qin X, Jiang H, Igboeli OC, Muzny D *et al* (2007). Paradoxical DNA repair and peroxide resistance gene conservation in Bacillus pumilus SAFR-032. *PLoS One* **2:** e928.

Grzymski JJ, Murray AE, Campbell BJ, Kaplarevic M, Gao GR, Lee C *et al* (2008). Metagenome analysis of an extreme microbial symbiosis reveals eurythermal adaptation and metabolic flexibility. *P Natl Acad Sci USA* **105:** 17516-17521.

Han CS, Xie G, Challacombe JF, Altherr MR, Bhotika SS, Brown N *et al* (2006). Pathogenomic sequence analysis of Bacillus cereus and Bacillus thuringiensis isolates closely related to Bacillus anthracis. *J Bacteriol* **188:** 3382-3390.

Hemme CL, Deng Y, Gentry TJ, Fields MW, Wu LY, Barua S *et al* (2010). Metagenomic insights into evolution of a heavy metal-contaminated groundwater microbial community. *Isme Journal* **4:** 660-672.

Hu X, Fan W, Han B, Liu H, Zheng D, Li Q *et al* (2008). Complete genome sequence of the mosquitocidal bacterium Bacillus sphaericus C3-41 and comparison with those of closely related Bacillus species. *J Bacteriol* **190:** 2892-2902.

Jung JY, Lee SH, Kim JM, Park MS, Bae JW, Hahn Y *et al* (2011). Metagenomic analysis of kimchi, a traditional Korean fermented food. *Appl Environ Microbiol* **77:** 2264-2274.

Junier P, Junier T, Podell S, Sims DR, Detter JC, Lykidis A *et al* (2010). The genome of the Gram-positive metal- and sulfate-reducing bacterium Desulfotomaculum reducens strain MI-1. *Environ Microbiol*.

Junier T, Zdobnov EM (2010). The Newick Utilities: High-throughput Phylogenetic tree Processing in the UNIX Shell. *Bioinformatics*.

Kalyuzhnaya MG, Lapidus A, Ivanova N, Copeland AC, McHardy AC, Szeto E *et al* (2008). High-resolution metagenomics targets specific functional types in complex microbial communities. *Nat Biotech* **26:** 1029-1034.

Katoh K, Kuma K, Toh H, Miyata T (2005). MAFFT version 5: improvement in accuracy of multiple sequence alignment. *Nucleic Acids Res* **33:** 511-518.

Konstantinidis KT, Braff J, Karl DM, DeLong EF (2009). Comparative Metagenomic Analysis of a Microbial Community Residing at a Depth of 4,000 Meters at Station ALOHA in the North Pacific Subtropical Gyre. *Applied and Environmental Microbiology* **75:** 5345-5355.

Kunin V, Raes J, Harris JK, Spear JR, Walker JJ, Ivanova N *et al* (2008). Millimeter-scale genetic gradients and community-level molecular convergence in a hypersaline microbial mat. *Mol Syst Biol* **4**.

Kunst F, Ogasawara N, Moszer I, Albertini AM, Alloni G, Azevedo V *et al* (1997). The complete genome sequence of the gram-positive bacterium Bacillus subtilis. *Nature* **390:** 249-256.

Kurokawa K, Itoh T, Kuwahara T, Oshima K, Toh H, Toyoda A *et al* (2007). Comparative metagenomics revealed commonly enriched gene sets in human gut microbiomes. *DNA Research* **14:** 169-181.

Martin HG, Ivanova N, Kunin V, Warnecke F, Barry KW, McHardy AC *et al* (2006). Metagenomic analysis of two enhanced biological phosphorus removal (EBPR) sludge communities. *Nat Biotech* **24:** 1263-1269.

Pope PB, Denman SE, Jones M, Tringe SG, Barry K, Malfatti SA *et al* (2010). Adaptation to herbivory by the Tammar wallaby includes bacterial and glycoside hydrolase profiles different from other herbivores. *P Natl Acad Sci USA* **107:** 14793-14798.

Rey MW, Ramaiya P, Nelson BA, Brody-Karpin SD, Zaretsky EJ, Tang M *et al* (2004). Complete genome sequence of the industrial bacterium Bacillus licheniformis and comparisons with closely related Bacillus species. *Genome Biol* **5:** R77.

Rice P, Longden I, Bleasby A (2000). EMBOSS: the European Molecular Biology Open Software Suite. *Trends Genet* **16:** 276-277.

Sattley WM, Madigan MT, Swingley WD, Cheung PC, Clocksin KM, Conrad AL *et al* (2008). The genome of Heliobacterium modesticaldum, a phototrophic representative of the Firmicutes containing the simplest photosynthetic apparatus. *J Bacteriol* **190:** 4687-4696.

Sebaihia M, Wren BW, Mullany P, Fairweather NF, Minton N, Stabler R *et al* (2006). The multidrug-resistant human pathogen Clostridium difficile has a highly mobile, mosaic genome. *Nature Genetics* **38:** 779-786.

Seedorf H, Fricke WF, Veith B, Bruggemann H, Liesegang H, Strittimatter A *et al* (2008). The genome of Clostridium kluyveri, a strict anaerobe with unique metabolic features. *Proceedings of the National Academy of Sciences of the United States of America* **105:** 2128-2133.

Shimizu T, Ohtani K, Hirakawa H, Ohshima K, Yamashita A, Shiba T *et al* (2002). Complete genome sequence of Clostridium perfringens, an anaerobic flesh-eater. *Proc Natl Acad Sci U S A* **99:** 996-1001.

Suen G, Scott JJ, Aylward FO, Adams SM, Tringe SG, Pinto-Tomas AA *et al* (2010). An Insect Herbivore Microbiome with High Plant Biomass-Degrading Capacity. *Plos Genetics* **6**.

Tringe SG, von Mering C, Kobayashi A, Salamov AA, Chen K, Chang HW *et al* (2005). Comparative Metagenomics of Microbial Communities. *Science* **308:** 554-557.

Turnbaugh PJ, Ley RE, Mahowald MA, Magrini V, Mardis ER, Gordon JI (2006). An obesity-associated gut microbiome with increased capacity for energy harvest. *Nature* **444:** 1027-1031.

Venter JC, Remington K, Heidelberg JF, Halpern AL, Rusch D, Eisen JA *et al* (2004). Environmental Genome Shotgun Sequencing of the Sargasso Sea. *Science* **304:** 66-74.

Warnecke F, Luginbuhl P, Ivanova N, Ghassemian M, Richardson TH, Stege JT *et al* (2007). Metagenomic and functional analysis of hindgut microbiota of a wood-feeding higher termite. *Nature* **450:** 560-U517.

Woyke T, Teeling H, Ivanova NN, Huntemann M, Richter M, Gloeckner FO *et al* (2006). Symbiosis insights through metagenomic analysis of a microbial consortium. *Nature* **443:** 950-955.

Wu M, Ren Q, Durkin AS, Daugherty SC, Brinkac LM, Dodson RJ *et al* (2005). Life in hot carbon monoxide: the complete genome sequence of Carboxydothermus hydrogenoformans Z-2901. *PLoS Genet* **1:** e65.
